# *Helicobacter pylori* infection perturbs iron homeostasis in gastric epithelial cells

**DOI:** 10.1371/journal.pone.0184026

**Published:** 2017-09-05

**Authors:** Sebastian E. Flores, Alan Aitchison, Andrew S. Day, Jacqueline I. Keenan

**Affiliations:** 1 Department of Surgery, University of Otago, Christchurch, New Zealand; 2 Department of Paediatrics, University of Otago, Christchurch, New Zealand; Lady Davis Institute for Medical Research, CANADA

## Abstract

The iron deficiency anaemia that often accompanies infection with *Helicobacter pylori* may reflect increased uptake of iron into gastric epithelial cells. Here we show an infection-associated increase in total intracellular iron levels was associated with the redistribution of the transferrin receptor from the cell cytosol to the cell surface, and with increased levels of ferritin, an intracellular iron storage protein that corresponded with a significant increase in lysosomal stores of labile iron. In contrast, the pool of cytosolic labile iron was significantly decreased in infected cells. These changes in intracellular iron distribution were associated with the uptake and trafficking of *H*. *pylori* through the cells, and enhanced in strains capable of expressing the *cagA* virulence gene. We speculate that degradation of lysosomal ferritin may facilitate *H*. *pylori* pathogenesis, in addition to contributing to bacterial persistence in the human stomach.

## Introduction

*Helicobacter pylori* inhabit the gastric mucosa of half the world’s population and without eradication therapy these bacteria may persist in this niche for the lifetime of the host. A proportion of those infected will develop gastric disease [1]. However, even in the absence of overt disease, infected individuals develop chronic gastritis [2]. There is also a growing awareness that chronic *H*. *pylori* infection may be associated with an increased risk of extragastric diseases that include host iron deficiency in humans [3] and in mice [4].

The link between *H*. *pylori* infection and the development of host iron deficiency is clearly illustrated by case studies that show individuals with idiopathic iron deficiency anaemia despite no apparent blood loss who are non-respondent to iron supplementation [5–7]. Remarkably, eradication of *H*. *pylori*, even without iron supplementation in some cases, restores iron status in these individuals [3]. How *H*. *pylori* is able to affect host iron homeostasis is not well understood but based on the observation of significantly less radioactive iron in red blood cells in *H*. *pylori*-infected individuals who are non-responsive to iron supplementation [6], it is possible that iron in circulation is simply diverted to the site of infection. If so, then any effect that *H*. *pylori* has on host iron stores is likely to first occur at the gastric epithelium, where these bacteria persist for a lifetime in untreated hosts [8]. Our recent observation of increased total iron in *H*. *pylori*-infected AGS gastric epithelial cells that is independent of the extracellular iron concentration supports this hypothesis [9].

Although largely non-invasive, a growing number of studies provide evidence that *H*. *pylori* can enter gastric epithelial cells *in vivo* [10] and *in vitro* [11,12], albeit at very low frequencies. There is also evidence that the number of bacteria entering the cells increases when the extracellular environment doesn’t support bacterial growth [12]. The idea that internalisation may provide *H*. *pylori* with a means to access an alternative source of iron has not yet been explored but there is evidence to support this idea. Detailed studies of the gram-negative bacterium, *Neisseria meningitidis* show that internalised bacteria are able to exploit intracellular ferritin, thereby providing a source of iron for the bacteria [13,14]. Moreover, possession of a similar mechanism by *H*. *pylori* would likely facilitate their persistence in the human stomach, given evidence that bacteria entering cell-associated compartments subsequently repopulate the extracellular environment [11].

The aims of this study were to determine how *H*. *pylori* bacteria affect the uptake, storage and/or distribution of iron in gastric epithelial cells, and to ascertain if changes in intracellular iron homeostasis correlate with bacterial uptake and trafficking through the cells. In addition, knockout strains were used to elucidate whether *H*. *pylori* uptake relates to CagA and VacA virulence factor expression, and if changes in intracellular iron homeostasis relate to the ability of bacteria to gain access to the cells. Our findings support the idea that persistent *H*. *pylori* colonisation of the gastric niche may relate to diversion of circulating iron into bacteria-containing compartments, and that expression of the CagA pathogenic determinant may convey an adaptive advantage with regards this aspect of infection.

## Materials and methods

### Reagents

Unless stated otherwise, all reagents were obtained from Sigma (St. Louis, MA). Bovine serum albumin (BSA), Dulbecco’s PBS (D-PBS), Trypsin-EDTA and Hoechst 33342 were all from Life Technologies (Mulgrave, VIC, Australia), ECL Plus Pierce Blotting Substrate, was purchased from Thermo Fisher Scientific (Auckland, New Zealand). The iron chelator salicylaldehyde isonicotinoyl hydrazone (SIH) was produced by Schiff base condensation from salicylaldehyde and isoniazid as previously described [15]. Briefly, equimolar solutions of salicylaldehyde (dissolved in one volume of ethanol) and Isoniazid (dissolved in 2 volumes of 25% (vol/vol) ethanol in water) were mixed and incubated in a steam bath for 20 min. The resultant solution was cooled, and filtered to recover a white-to-yellowish powder that was dried at room temperature before being recrystallized with ethanol to remove impurities. Mass spectrometry of the powder dissolved in DMSO showed a compound with a molecular weight of 241g/mol and ~93.3% of purity.

### Bacterial strains and culture

*H*. *pylori* strain 60190 (ATCC 49503), a well characterized clinical isolate that is *cagA* positive [16] and has a *vacA* s1m1 genotype [17], was used for this study, along with two isogenic mutants derived from this strain by insertion of a kanamycin resistance cassette to disrupt the *cagA* [18] and *vacA* [19] genes, respectively (kindly provided by Drs Rick Peek and Tim Cover, Vanderbilt University School of Medicine, USA). The bacteria were routinely cultured on Columbia sheep blood agar plates (Fort Richard, Auckland, NZ) in a microaerophilic atmosphere at 37°C for 2 to 3 days. Prior to co-culture with AGS cells, *H*. *pylori* were resuspended in RPMI-1640 medium (Invitrogen), supplemented with 10% (v/v) heat-inactivated foetal bovine serum (FBS, Invitrogen) and quantitated by density, based on a standard curve measured at an absorbance of 620nm (SpectraMax® 190, Molecular devices, Sunnyvale, CA, USA). An absorbance of 0.2 was determined to be approximately 1 x 10^8^ colony forming units (CFUs) per ml. Bacterial addition to cell cultures was at a multiplicity of infection (MOI) of 10:1. The Vybrant dye DiO (Life Technologies, Eugene, OR) was used to label *H*. *pylori*. Washed bacteria were resuspended in 200 μl of RPMI media with the dye added at a final concentration of 100 μM. The bacteria were incubated in the dye for 20 min at 37°C, washed twice with D-PBS to remove unbound dye and quantitated by density.

### Cell culture and treatment

The AGS human gastric adenocarcinoma cell line (ATCC CRL 1739) was cultured in RPMI-1640 medium, supplemented with 10% (v/v) heat-inactivated FBS and 1% (v/v) penicillin-streptomycin supplement (Life Technologies, Auckland, NZ). Cells were cultured at 37°C with 5% CO_2_. Antibiotics were omitted for assays that included co-culture of cells with live bacteria.

### RNA isolation and cDNA synthesis

AGS cells (1 x 10^5^) were cultured in 12-well plates for 72 h. The medium was replaced in each well and the cells were exposed (or not) to *H*. *pylori* (MOI of 10:1) or 100 μm iron sulphate for 30 min, 3 h and 12 h before the total RNA was extracted (RNeasy Mini Kit, Qiagen, Hilden, Germany). Total RNA content was measured using a NanoDrop spectrophotometer (Nanodrop Technologies, Montanin, DE). Samples were treated with DNase I at 37°C for 30 min, followed by 10 min at 75°C and 5–10 min at 4°C. First strand cDNA was synthesised from total RNA (1 μg) using qScript XLT cDNA Supermix (Quanta Biosciences, Beverly, MA).

### Real-time (RT) quantitative polymerase chain reaction (qPCR)

RT-qPCR was used to determine whether *H*. *pylori* infection of AGS cells was associated with an increase in TfR and/or ferritin gene expression over time. All qPCRs were performed in 10 μl reaction mixture containing 10 ng/μl of cDNA, 5 μl of PerfeCTa Fastmix (Quanta Biosciences), forward and reverse primers (S1 Table), and nuclease-free water. Reactions were performed in triplicate in a LightCycler 480 (Roche Diagnostics, Pleasanton, CA). The cycling conditions consisted an initial polymerase activation step at 95 ^o^C for 2 min, followed by 40 cycles of 95 ^o^C for 15 s, 55 ^o^C for 15 s and 68 ^o^C for 20 s. The specificity of amplification was verified by melting curve analysis (60–95 ^o^C). For each sample the Ct value (the fluorescent point at which the reactions are compared) was fitted to a standard curve consisting of five serial dilution points (in triplicate) of purified DNA template and a no-template control. TfR and heavy (H-) chain ferritin mRNA expression was calculated using the 2^-ΔΔCt^ method normalised against hypoxanthine phosphoribosyltransferase 1 (HPRT) as the endogenous RNA control.

### Preparation of whole cell lysates for immunoblotting

AGS cells (1 x 10^5^) were cultured in 12-well plates for 72 h. The medium was replaced in each well and the cells were exposed (or not) to *H*. *pylori* (MOI of 10:1) or 100 μm iron sulphate for 30 min, 3 h and 12 h before being lysed. Briefly, the culture medium was removed and the cells washed four time (D-PBS) before the cells were lifted with 2mM EDTA to avoid trypsin-mediated cleavage of cell membrane receptors [20]. The washed cell pellet was digested with RIPA buffer (50 mM Tris HCl pH 7.5, 100 mM NaCl, 5 mM EDTA pH 7.5, 1% (v/v) Nonidet P-40, 0.2% (w/v) SDS, 0.5% (w/v) sodium deoxycholate, pH 7.5, 1 mM sodium vanadate, 1 mM phenylmethanesulfonyl fluoride, protease inhibitor (cOmplete Mini, Roche, Basel, Switzerland) for 20 min on ice, with vortexing every 5 min to facilitate cell lysis. The lysates were centrifuged at 14000 x *g* for 30 min at 4°C to remove remaining cellular debris before being assayed for protein content and stored at -20°C.

### Immunoblot analysis of transferrin receptor and ferritin expression in AGS cells

AGS cell lysates (10 μg protein) separated by discontinuous SDS-PAGE under reducing conditions on a 10% acrylamide gel were transferred to a polyvinylidene difluoride (PVDF) membrane (GE Healthcare, Buckinghamshire, UK) and the membrane blocked for 60 min at room temperature before being probed overnight at 4^oC^. The primary antibodies were anti-human transferrin receptor antibody (Invitrogen) or anti-human H-ferritin antibody (Abcam, Cambridge, MA), with binding detected using horse radish peroxidase (HRP)-conjugated polyclonal goat anti-mouse IgG (Dako, Agilent, Santa Clara, CA) or rabbit anti-goat IgG (Sigma, St Louis, MO), respectively. The blocking solution was 5% (w/v) non-fat milk in Tris buffered saline (TBS; 20 mM Tris-HCl pH 7.5, 140 mM NaCl) containing 0.1% (v/v) Tween-20 (TBS-T), and the antibodies were diluted in 2.5% (w/v) non-fat milk in TBS-T. The membrane was developed with a chemiluminescent substrate (ECL Plus Pierce Blotting Substrate, Thermo Fisher Scientific) and the blot photographed using a Uvitec imager (Uvitec, Cambridge, UK). Membranes probed for ferritin were stripped with buffer containing 62.5mM Tris-HCl pH 6.8, 2% (w/v) SDS and 100 mM β-mercaptoethanol for 50 minutes at 50°C in a hybridization oven, washed 3 times with TBS-T and then re-probed using an antibody directed against GAPDH. Densitometry analysis of signal intensity was carried out using UVIBand software (Uvitec), with the mean pixel intensities (MPIs) of the staining in infected and uninfected cells lysates calculated from three independent experiments.

## Fluorescence microscopy

AGS cells (8 x 10^4^) grown on glass coverslips in six-well plates for 72 h were washed with PBS to remove antibiotics before infection (or not) with *H*. *pylori* (MOI 10:1) or co-culture with 100 μm ferric sulphate. After 15 h the cells were washed (D-PBS) and fixed (4% [v/v] formaldehyde in PIPES buffer (60mM PIPES, pH 6.8)) before being permeabilized (0.1% (v/v) Triton X-100 in PBS, 15 min) or left intact. After three PIPES buffer washes the cells were blocked with PIPES buffer, 5% (w/v) bovine serum albumin) for 1 h before overnight incubation at 4°C in primary antibody (anti-human TfR or anti-human H-ferritin). After another 3 washes, the coverslips were incubated for 90 min at 37°C in secondary antibodies; Alexa Fluor 488-conjugated anti-mouse IgG (TfR) or biotin-conjugated anti-goat IgG antibody followed by Phycoerythrin-conjugated streptavidin to detect ferritin. All antibodies were diluted in PIPES buffer, 2.5% (v/v) FBS. Cell nuclei were counterstained with Hoechst 33342 (0.25 μg/ml in PIPES) for 20 min, with Texas Red-conjugated phalloidin (1U/ml, Invitrogen) used in some instances to highlight the cytoplasm. The coverslips were mounted in antifade reagent (ProLong® gold; Invitrogen). Slides were examined using a Zeiss Axioimager Z1 microscope with a 40x EC Plan Neofluar NA 1.4 objective and ApotomeTM structure illumination system. Image stacks of 30 focal planes were obtained using an Axiocam MRm camera, coupled to Axiovision software (Zeiss, Oberkochen, Germany). ImageJ software from the NIH [21] was used to mount Z-projections using the maximum fluorescent intensity, keeping brightness and contrast constant in all images from the same set, to facilitate visual comparison.

To show that intracellular *H*. *pylori* traffic to acidified compartments, cells infected with DiO-labelled *H*. *pylori* were treated with 100 nM LysoTracker Red (Invitrogen, Carlsbad, CA) in medium during the last two hours of incubation. The cells were washed, fixed, mounted with ProLong® gold and evidence of DiO-labelled fluorescent bacteria colocalization with LysoTracker Red was determined using fluorescence microscopy (as above).

### Lysosomal iron levels

The total intracellular labile iron was estimated by the sulphide-silver method [22]. Briefly, AGS cells (8 x 10^4^) were grown on coverslips for 72 h prior to the addition (or not) of *H*. *pylori* (MOI 10:1) for 15 h. The cells were washed 4 times with D-PBS to remove non-adherent bacteria, fixed with 2% (v/v) glutaraldehyde (in 0.1 M cacodylate buffer containing 0.1 M sucrose, pH 7.2) for 2 hours at room temperature, washed extensively with ultrapure water and then exposed to 1% (v/v) ammonium sulphide (in 70% ethanol, pH 9) for 15 min. After another 4 washes with ultrapure water, staining of cellular lysosomal iron was developed by coating each coverslip with a physical colloid-protected developer (containing silver and hydroquinone) for 30 min at room temperature (in the dark), with care taken to develop each coverslip for exactly the same time. The reaction was stopped by washing coverslips thoroughly with ultrapure water to remove the developer. Samples were then dehydrated on an ethanol-graded series and mounted with Canada balsam and allowed to dry for at least 5 days in the dark before images were captured using light microscopy (Axioimager Z1 microscope). Densitometry analysis on ImageJ software was used to quantify the staining. Briefly, five sets of images (from 3 independent experiments) were analysed by obtaining a mean pixel intensity (MPI) for each cell present within an image. The mean pixel intensity of the infected cells (expressed as a percentage of the values obtained from uninfected cells), number of analysed cells and standard deviation for each image were then used to express differences in staining intensities between different conditions (S1 Fig).

### Measuring the cytosolic labile iron pool

The calcein-AM assay was used to assess the amount of intracellular iron present as part of the cytoplasmic labile iron pool (cLIP) in AGS cells [23]. Briefly, the cells (1 x 10^5^) were cultured for 72 h before the addition (or not) of *H*. *pylori* (MOI 10:1) for 15 h. The cells were washed 4 times with D-PBS before incubation with 0.15 μM calcein-AM (in PBS) for 15 min at room temperature. Cells were lifted with trypsin-EDTA, and resuspended to 1 x 10^6^ cells per ml in diluent containing D-PBS with 10% (v/v) FBS, 0.2% ethanol and 0.02% dimethyl sulphoxide (DMSO). 150 μl aliquots of the cell suspensions (containing approximately 1.5 x 10^5^ cells) were transferred (in triplicate) to a 96-well flat bottomed plate with black walls (Greiner Bio-one, Monroe NC), and the basal fluorescence was read (at 492 nm and 520 nm for excitation and emission, respectively) using a Polarstar Galaxy fluorescent plate reader (BMG Labtech, Ortenberg, Germany) before 150 μl of SIH (200 μM, in diluent) was added to each well for 2 min. Fluorescence was re-read and ΔF was determined as a percentage of the basal fluorescence, with results expressed as a percentage of uninfected cells.

### Phagosome extraction and dot blotting

This assay was performed to determine if internalised *H pylori* traffic via the endosomal pathway. AGS cells exposed (or not) to *H*. *pylori* (MOI 10:1) for 15 h were washed five times with D-PBS to remove non-adherent bacteria (as above), before the cells were lifted (trypsin-EDTA, 10 min, 37°C), washed (D-PBS) and lysed by sonication (in 250 mM sucrose, 0.5 mM EGTA, 20 mM HEPES-KOH, pH 7.2), as reported elsewhere [24]. Unbroken cells and whole nuclei were removed (300 x *g* at 4°C for 3 min) before the lysates were centrifuged through a sucrose density gradient (65% to 10%) at 100,000 x *g* for 1 h at 4°C. Ten fractions were collected and the presence of *H*. *pylori*, ferritin and/or Rab7 was determined by dot blotting replicates of each of the 10 fractions transferred to PVDF membranes. Each membrane was blocked for 1 h at RT in 5% (w/v) non-fat milk in TBS-T before being individually probed overnight at 4°C with antibodies to H-ferritin, *H*. *pylori* Lpp20 [25] or Rab7 [24], followed by incubation in HRP-conjugated secondary antibody for 90 min at RT. Antibodies were diluted in 2% non-fat milk (w/v) in TBS-T, the membranes were developed with a chemiluminescent substrate (ECL Plus Pierce Blotting Substrate, Thermo Fisher Scientific), and the signal visualised using a Uvitec imager (Uvitec).

### Flow cytometry

Flow cytometry was used to confirm that *H*. *pylori* is internalised into AGS cells. AGS cells (1 x 10^5^) were cultured in 12-well plates for 72 h. The medium was replaced in each well and the cells were exposed (or not) to DiO-labelled *H*. *pylori* (MOI 10:1) for 15 h. Non-adherent bacteria were removed by washing individual wells five times with D-PBS. The cells were lifted and re-suspended in 5% (v/v) FBS in D-PBS, and fluorescence measurements were made using a flow cytometer (Cytomics FC 500 MPL; Beckman Coulter) and CXP software (Beckman Coulter). Ten thousand events were collected for each sample, with size stratification used to exclude remaining non-adherent bacteria. Mean fluorescent intensity (MFI) values of cells incubated in the absence of bacteria were subtracted from values of bacterium-treated cells. To determine the proportion of internalised bacteria, fluorescence was measured after the addition of trypan blue (final concentration, 0.05%) to quench extracellular bacterial fluorescence [24].

### Gentamycin protection assay

This assay was performed to determine at what frequency *H*. *pylori* is internalised into AGS cells. AGS cells (1 x 10^5^) were cultured in 12-well plates for 72 h. The medium was replaced in each well and the cells were exposed (or not) to *H*. *pylori* (MOI 10:1) for 15 h. Non-adherent bacteria were removed by washing individual wells with D-PBS, then 100 μg/ml gentamicin (in medium) was added and the cells were incubated for 2 h at 37°C. The cells were then washed five times with D-PBS before lysis buffer (0.1% [v/v] Triton X-100 in PBS) was added to each well for 10 min at 37°C. Bacteria in the lysate were quantified by serial dilutions on blood agar plates incubated for three days at 37°C in a microaerophilic environment (12).

### Statistical analysis

Results are presented as the ± SE of the means of at least three independent experiments. Data were analysed by ANOVA followed by Tukey’s post hoc test (for multiple comparisons between more than two groups) or non-parametric t-test, as appropriate. If p < 0.05, the differences were considered to be statistically significant. Statistics were calculated with GraphPad Prism software (version 6.00).

## Results

### Ferritin increase in *H*. *pylori*-infected AGS cells does not reflect cell-associated bacteria

An infection-associated increase in total iron levels seen when *H*. *pylori* are added to cultured gastric AGS cells [9], which was not a result of bacterial association with the cells (S2 Fig), suggested that *H*. *pylori* may perturb iron uptake and/or storage mechanisms involved in maintaining cellular iron homeostasis.

**Fig 1:**
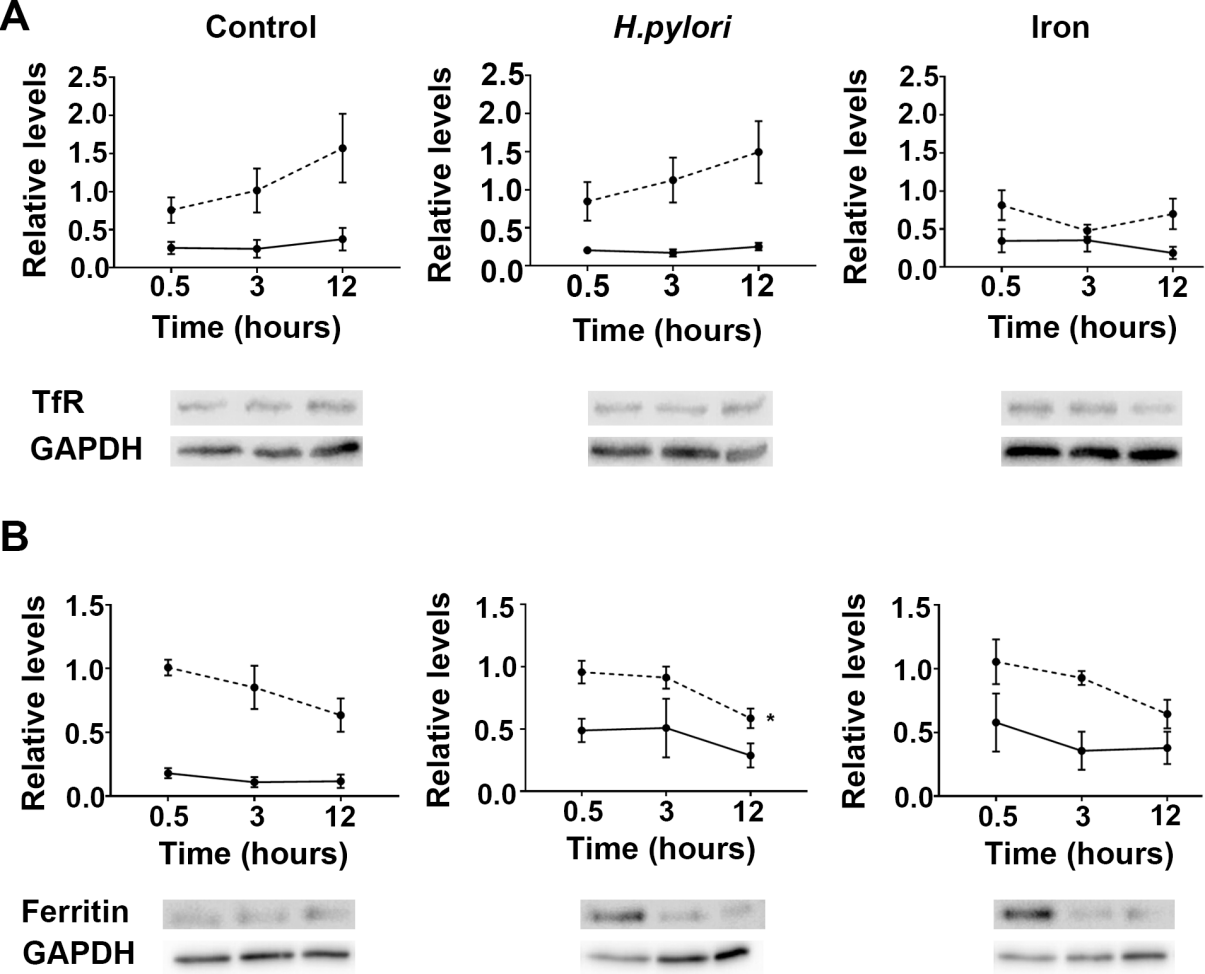
Quantification of transferrin receptor mRNA and protein in AGS cells infected with *H*. *pylori*. Cells were infected with wild type *H*. *pylori* strain 60190 (MOI, 10:1) or supplemented with 100 μm iron sulphate, with (A) transferrin receptor (TfR) and (B) H-ferritin mRNA (dotted line) and protein levels (solid line) quantitated throughout 12 h of infection. Levels are expressed as the amount of RNA and protein relative to endogenous controls HPRT and GAPDH, respectively, with representative immunoblots shown below the graphs. Data points for mRNA levels represent the average of three experiments each done in triplicate, whereas data points for protein represent ±SEMs for three independent immunoblotting experiments. *, levels over time are statistically different (P<0.05), as determined by one-way ANOVA with Tukey’s test for multiple comparisons.

The transferrin receptor (TfR) is a protein of approximately 95 kDa that has an established role in the uptake of iron-loaded transferrin into a variety of cell types including AGS cells [26] whereas ferritin is recognized as an intracellular iron storage protein [27]. RT-PCR revealed an increase in TfR gene expression in uninfected and *H*. *pylori*-infected cells over time that was not discernibly different between the two conditions (Fig 1A; broken line; left and middle). Likewise, immunoblotting of cell lysates revealed that infection had no discernible effect on TfR protein expression when compared to uninfected cells (Fig 1A; solid line; left and middle). In contrast, the expression of this gene was notably reduced in AGS cells supplemented with iron sulphate whereas TfR protein levels were increased (Fig 1A; right) compared to infected and uninfected cells. Collectively, these findings suggest that unlike iron, *H*. *pylori* infection does not change the expression of TfR in AGS cells.

RT-PCR revealed that heavy (H) chain ferritin expression in *H*. *pylori-*infected cells fell significantly over the 12 h time period (P<0.05; Fig 1B). A corresponding decrease in H-ferritin gene expression over time was also observed in uninfected and iron-supplemented cells (Fig 1B) but these changes failed to reach significance. In contrast, H-ferritin protein expression was increased in both infected and iron sulphate-supplemented cells when cell lysates were immunoblotted and compared to lysates of uninfected cells (Fig 1B). Moreover, 30 min after AGS cells were infected with *H*. *pylori* H-ferritin protein levels were significantly increased compared to the uninfected cells (P<0.05; unpaired t-test). This increase was not sustained and over time H-ferritin protein levels fell in both the infected and iron-supplemented cells. However, levels remained notably higher than those observed in the uninfected cells at 12 h (Fig 1B).

**Fig 2:**
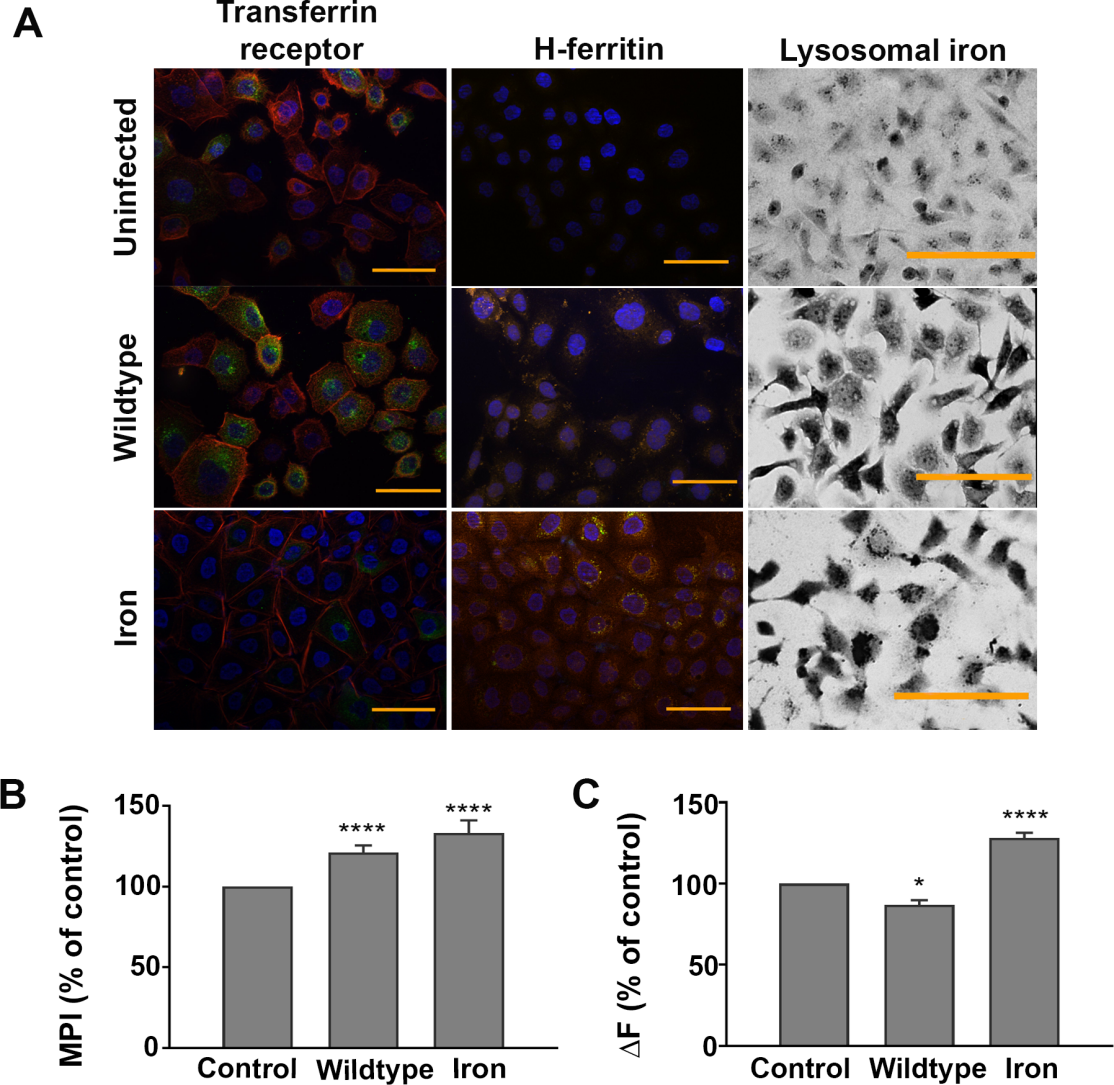
Distribution of transferrin receptor and H-ferritin in *H*. *pylori*-infected AGS cells. AGS cells infected with *H*. *pylori* strain 60190 (MOI, 10:1) or supplemented with iron sulphate (100 μm) for 15 h were fixed and stained with Alexa Fluor 488-labelled transferrin receptor (green), Texas Red-conjugated phalloidin-stained actin (red) and Hoechst 33342-stained nuclei (blue) in non-permeabilized cells; Streptavidin-Phycoerythrin-stained H-ferritin (orange) and Hoechst 33342-stained nuclei (blue) in Triton X-100 permeabilized cells, and lysosomal iron using sulphide-silver (left, middle and right columns, respectively). The images, which are representative of three independent experiments, denote cells with no bacteria (Uninfected), infected with *H*. *pylori* strain 60190 (Wildtype) or supplemented with 100 μm iron sulphate (Iron) for 15 h. All images were taken with the same exposure time for each channel. Bar = 50μm. **(B)** The mean pixel intensity (MPI) of sulphide-silver stained AGS cells following infection with *H*. *pylori* or supplemented with iron sulphate calculated using ImageJ software, with levels of lysosomal iron (evidenced by MPI) expressed as a percentage of that observed in uninfected (Ctrl) cells. **(C)** *H*. *pylori-*infected and iron-supplemented AGS cells treated with calcein, with and without the addition of SIH to measure the cytosolic labile iron pool. Results, which show an increase in fluorescence (ΔF) in infected and iron-supplemented cells, are presented as a percentage of uninfected cells. **(B)** Results are ± SEM of five images taken from three independent experiments and **(C)**, ± SEM of 3 independent repeats. *, ****, results are statistically different from those of untreated controls (P<0.05 and <0.0001, respectively), as determined by one-way ANOVA with Tukey’s test for multiple comparisons.

Immunofluorescence microscopy of uninfected, infected and iron-supplemented AGS cells permeabilised before staining revealed no obvious difference in the localization and/or intensity of TfR staining across these three conditions. In contrast, when similarly treated cells were left intact (non-permeabilised) and stained, it was apparent that the surface-associated staining of TfR in the uninfected and iron-supplemented AGS cells was similar, and notably less than that seen on AGS cells infected with *H*. *pylori* (Fig 2, left column). Microscopy also supported the observed increase in H-ferritin protein expression with evidence of cytosolic distribution of ferritin in infected and iron-supplemented cells. Collectively these findings suggest a mechanism of iron uptake by *H*. *pylori*-infected AGS cells that involves redistribution of the TfR receptor to the cell surface and storage of this iron associated with ferritin.

### Cellular iron homeostasis is perturbed in *H*. *pylori*-infected AGS cells

Evidence of punctate staining in infected and iron-supplemented AGS cells (Fig 2; middle column) suggested the accumulation of ferritin was compartmentalized. This was confirmed by sulphide-silver staining of the cells, which revealed labile iron-rich lysosomal compartments within the *H*. *pylori*-infected and iron-supplemented AGS cells (Fig 2; right column). In contrast, H-ferritin and sulphide-silver staining of uninfected AGS cells was minimal, with staining evenly distributed throughout the cytosol (Fig 2; top row). Densitometric analysis of the images from three independent experiments confirmed there was a significant increase in staining intensity when *H*. *pylori* strain 60190 was added to AGS cells (121.2% ± 4.5) and the results normalized to uninfected cells (100%, P<0.0001; Fig 2B). A similar accumulation of iron-rich lysosomes was also observed in silver-sulphide stained AGS cells following incubation with 100 μm iron sulphate (133.2% ± 7.8 of uninfected control) and again, this difference was significant (P<0.0001; Fig 2B). However, whereas the addition of iron sulphate also significantly increased the cytosolic labile iron pool (cLIP) in cells (128.2% ± 3.1), *H*. *pylori* infection had the opposite effect (86.88% ± 2.95) when compared to uninfected cells (P<0.0001 & <0.01, respectively; Fig 2C). Accordingly, the finding of a decreased cLIP suggested that cellular iron homeostasis is perturbed in *H*. *pylori*-infected cells.

### *H*. *pylori* entry into AGS cells

**Fig 3:**
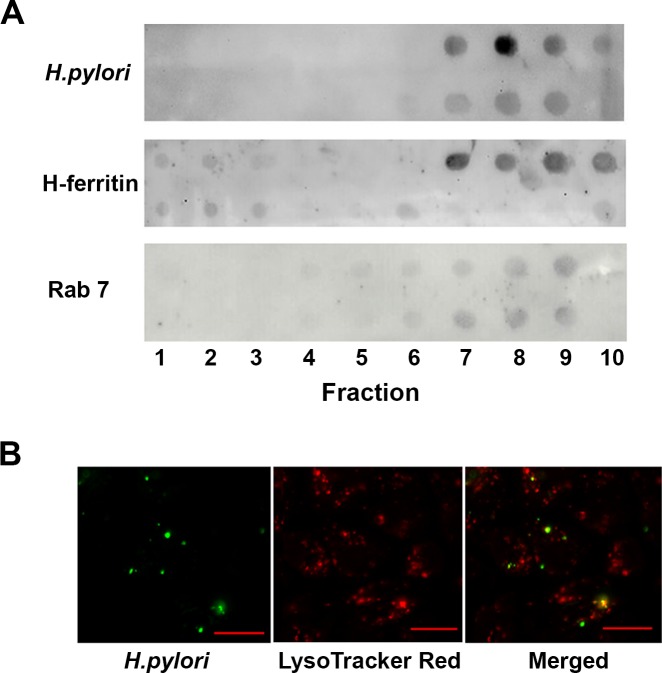
*H*. *pylori* co-localise with ferritin in Rab7-rich compartments within AGS cells. **(A)** Phagosomes were collected from lysed AGS cells following infection with *H*. *pylori* strain 60190 (MOI, 10:1) for 15 h (top) or not (bottom) by sucrose density gradient centrifugation. Ten (top down) fractions were blotted onto PVDF membrane and probed with antibodies to *H*. *pylori* Lpp20, H-ferritin and Rab7. Antibody binding was visualised using a chemiluminescent detection of HRP-conjugated secondary antibody binding. Images are representative for three independent experiments. **(B)** AGS cells infected with DiO-labelled *H*. *pylori* strain 60190 and stained with LysoTracker Red are shown individually and in a merged image. Bar = 20μm.

Phagosomes extracted from infected and uninfected AGS cells probed with a monoclonal antibody specific for Lpp20, an *H*. *pylori* outer membrane-associated lipoprotein [25], suggested that internalized bacteria traffic to Rab7-enriched compartments within the cells that also stained positive for H-ferritin (Fig 3A). Rab7 recruitment to phagosomes occurs before the final step in phagosome maturation, which involves binding and fusion of secondary lysosomes and subsequent acidification of the phagosome [28]. DiO-labelled *H*. *pylori* added to AGS cells co-stained with LysoTracker Red, a red-fluorescent dye used for labeling and tracking acidic organelles in live cells, showed evidence of internalized bacteria in acidic organelles (Fig 3B). Collectively these findings suggest that the uptake of *H*. *pylori* may be associated with the observation of perturbed cellular iron homeostasis, and raised the question of whether the CagA and/or VacA virulence factors are involved.

### CagA increases iron uptake and lysosomal storage in *H*. *pylori*-infected AGS cells

**Fig 4:**
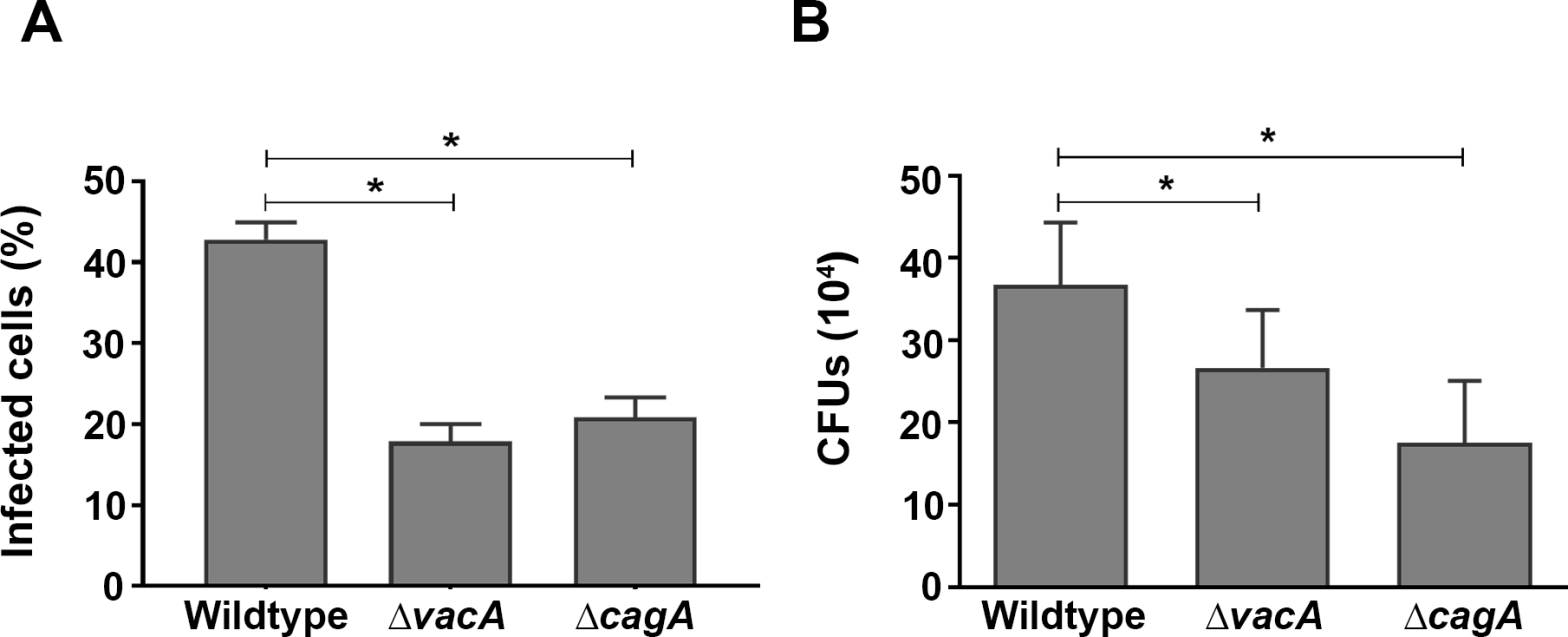
*H*. *pylori* uptake by AGS cells. **(A)** Flow cytometry was used to assess internalization of DiO-labelled bacteria following infection of AGS cells with *H*. *pylori* (MOI, 10:1) for 15 h. **(B)** Infected cells were treated with gentamycin for 2 h to kill any remaining extracellular bacteria. Quantitative assessment of viable *H*. *pylori* in AGS cells was by serial dilutions of cells lysates. Results are ± SEM of three independent experiments. *, results are statistically different from those of cells infected with H. pylori wildtype strain 60190 (P<0.05), as determined by one-way ANOVA with Tukey’s test for multiple comparisons.

Isogenic mutants where the *cagA* (Δ *cagA*) and *vacA* (Δ *vacA*) genes were respectively inactivated in wildtype strain 60190 [18,19] were used to test this hypothesis. Flow cytometry revealed that *H*. *pylori* are internalized into AGS cells, that uptake was greatest when both *cagA* and *vacA* genes were present, and that ablation of the *cagA* and *vacA* genes significantly reduced the number of infected AGS cells following co-culture with the bacteria (P<0.05; Fig 4A). This finding was confirmed by gentamicin protection assay that revealed approximately 4% of the 1 x10^7^ *H*. *pylori* 60190 added to AGS were recovered from inside the cells following 15 h incubation (3.7 x 10^5^ CFUs ± 7.6 across four independent experiments), and that ablation of the *cagA* or *vacA* genes had a significant effect on the number of viable bacteria recovered from infected AGS cells (p<0.05; Fig 4B).

**Fig 5:**
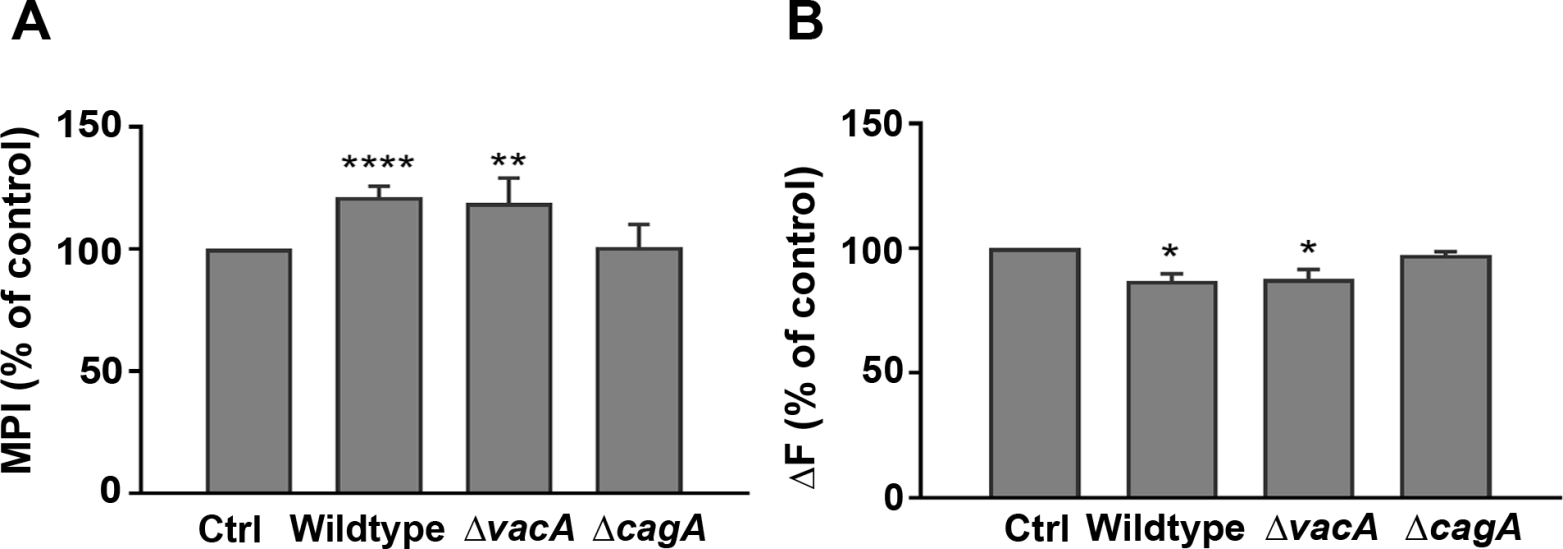
Labile iron pool in AGS cells infected with *H*. *pylori* CagA and VacA mutants. AGS cells infected with *H*. *pylori* (MOI, 10:1) for 15 h were **(A)** fixed and stained with sulphide-silver to detect lysosomal iron or **(B)**, treated with calcein with and without the addition of SIH to measure the cytosolic labile iron pool. **(A)** The mean pixel intensity (MPI) of each image was calculated using ImageJ software and evidence of lysosomal iron in infected cells (indicated by MPI) was expressed as a percentage of that observed in uninfected (Ctrl) cells. **(B)** Results reflect the increase in fluorescence (ΔF) in cells infected with the wildtype, and VacA and CagA mutant strains of *H*. *pylori* strain 60190 presented as a percentage of uninfected cells. **(A)** Results are ± SEM of five images taken from three independent experiments and **(B)**, ± SEM of 3 independent repeats. *, **, ****, results are statistically different from those of untreated controls (P<0.05, <0.01 and <0.0001, respectively), as determined by one-way ANOVA with Tukey’s test for multiple comparisons.

TfR expression at the surface of AGS cells infected with the CagA mutant strain (S3 Fig; left column) was not discernibly different from that observed in uninfected cells (Fig 2), and there was notably less evidence of intracellular ferritin (middle) and lysosomal iron (right) in these cells when compared to cells infected with VacA mutant strain of *H*. *pylori* (S3 Fig). In contrast, expression of the TfR and ferritin staining, and the intensity of silver sulphide staining of AGS cells infected with the *vacA* mutant (S3 Fig) closely resembled the pattern and intensity of staining of cells infected with wildtype strain 60190 (Fig 2). Moreover, densitometric analysis of the intensity of sulphide-silver staining confirmed there was a higher pixel intensity in those cells infected with the two CagA+ strains (wildtype and VacA mutant; Fig 5A) when compared to uninfected cells (P<0.01 and <0.0001, for VacA mutant and wildtype, respectively). In contrast, cells infected with the *H*. *pylori* CagA+ strains had a significantly lower cLIP (P<0.05 for both wildtype and VacA mutant strains) when compared to uninfected cells (Fig 5B). Taken together, these findings suggest that increased levels of lysosomal labile iron are found in cells infected with CagA+ strains of *H*. *pylori*.

## Discussion

Iron is an essential micronutrient for both animals and microorganisms due to its capacity to act as a cofactor for enzymes involved in oxygen transport, DNA synthesis and electron transport. As a response to infection, an iron sequestering mechanism has developed in mammals that reduces the growth of infecting pathogens by increasing ferritin (intracellular and circulating) and reducing circulating transferrin [29]. To avoid this response, human pathogens have evolved alternative mechanisms to obtain iron from their host, enabling their survival. This is exemplified by persistent *H*. *pylori* infection of the gastric mucosa. These bacteria are associated with the development of iron deficiency in humans [3] and mice [4], and the observation that diminished iron stores in mice are further lowered by concurrent infection indicates that *H*. *pylori* competes successfully with the host for available iron [4]. In turn, mounting evidence suggests that iron deficiency accelerates *H*. *pylori*-induced carcinogenesis in rodents and humans [30]. However, how *H*. *pylori* acquires host iron remains unclear.

*H*. *pylori* uptake by AGS cells is associated with an increase in total cellular iron content that is independent of extracellular concentrations of iron and does not reflect cell-associated bacteria [9]. Iron levels inside cells are governed by uptake, usage, storage and export processes, with both the transferrin receptor (TfR) and ferritin playing a major role in maintaining iron homeostasis in cells whose principal function is not systemic iron uptake or storage. The expression of these two intracellular proteins is controlled by two iron regulatory proteins (IRP1 and IRP2) that act as sensors attaching to specific sequences (iron responsive elements, IREs) in the 3’ and 5’ non-coding regions of TfR and ferritin mRNAs, respectively, when cLIP levels are low [31]. Thus, in iron-rich cells, IRP activity is decreased, TfR synthesis reduced while H- and L-ferritin production is enhanced, exemplified here by the response of AGS cells to iron supplementation.

*H*. *pylori* had no discernible effect on TfR expression in this study or in an unrelated prior study that used the Madin-Darby canine kidney (MDCK) cell line [32]. However, the observation of an infection-associated redistribution of the TfR receptor from the cytosol to the surface of AGS cells, coupled with an increase in transferrin uptake by infected MDCK cells [32] and a significant increase in ferritin-rich intracellular compartments in infected AGS cells (this study) strengthens the hypothesis that these bacteria have the potential to perturb cellular iron homeostasis. While they are normally considered non-invasive, the observation of that small numbers of bacteria are taken up and traffic to intracellular compartments inside AGS cells is in line with other studies [11,12,33,34]. Unrelated studies that demonstrate the presence of LAMP-1 [33] and absence of cathepsin D [34] in these bacteria-rich compartments, together with the finding that the bacteria-rich phagosomes are ferritin-rich, suggests they are likely to be post endosomal hybrids of late endosomes and lysosomes [35], an observation that is strengthened by the finding of internalised bacteria in acidic compartments, as evidenced by LysoTracker Red staining.

The decrease in the level of the cytosolic labile iron pool (cLIP) in the infected cells adds weight to the hypothesis that *H*. *pylori* may perturb intracellular iron homeostasis. It remains to be determined whether this occurs at the level of post-transcriptional regulation of the IRP/IRE system and/or an acute phase response to infection. It also remains to be determined whether *H*. *pylori* have developed the means to subvert the trafficking of iron released from ferritin when lysosomal levels are high. Normally this iron, which is released by proteolytic degradation, is delivered back to the cytosol through ferrous iron transporters resulting in a concomitant increase in the cLIP [36], as seen in A431 cells, a human endocervical cell line, following infection with *Neisseria meningitidis* [14]. That *H*. *pylori* do not effect a similar response suggests that they have evolved a unique means to subvert the iron released from ferritin to their advantage.

Intriguingly, this effect is not seen when *H*. *pylori* outer membrane vesicles (OMV) that are shed constitutively from the bacterial surface [37] are added to AGS cells. *In vitro* studies show the vesicles enter AGS cells [38] and affect the intracellular distribution of ferric iron [39]. Specifically, strain 60190 OMV added to AGS cells results in the movement of ferric iron from intracellular compartments to the cytosol that is associated with increased oxidative stress and DNA damage [39]. Thus, the observation that redox-active lysosomal iron is not delivered back to the cytosol in cells infected with viable *H*. *pylori* suggests that these bacteria, by disrupting delivery of this iron back to the cytosol, may have evolved a unique mechanism to protect themselves from oxidative damage [40]. This would fit with the observation that *H*. *pylori* leave these intracellular compartments to repopulate the cell surface [11]. Future experiments will reveal whether *H*. *pylori* can access the lysosome-derived iron intracellularly, or if the released iron is discharged to the extracellular space by the same route that the bacterium uses to exit the cell [41].

*H*. *pylori* strain 60190 produces two well described virulence factors, the cytotoxin-associated gene A (CagA) and the vacuolating cytotoxin A (VacA) proteins, both of which are associated with increased pathogenicity following infection [42,43]. In this study CagA and VacA were not essential for *H*. *pylori* entry into AGS over the course of the experiment. However, notably more of the strain 60190 bacteria that express both virulence factors were internalized by AGS cells when compared cells exposed to strain 60190 isogenic mutants lacking expression of VacA, and this difference was even more evident when wildtype bacteria were compared with the CagA mutant. Cultured AGS cells are a model of non-polarized gastric epithelia and CagA is thought to facilitate receptor-mediated endocytosis of *H*. *pylori* into these cells [33]. A CagA mutant also fails to colonise the apical cell surface in polarised epithelia but this is overcome when the bacteria have access to the basolateral cell surface [44]. Whereas this highlights the physiological differences between the two models used for these respective studies, the finding that the presence of CagA protein correlated strongly with disturbances in AGS cell iron homeostasis in response to *H*. *pylori* infection is in agreement with Tan’s study, which shows a two-fold increase in transferrin internalization from the basolateral side of MDCK cells after 30 minutes of exposure to holo-transferrin is dependent on the intracellular phosphorylation of CagA and does not represent a difference in total expression of the transferrin receptor [32]. Whether or not VacA has a role in this process remains to be determined. This is because our finding of notably fewer CagA mutant bacteria inside AGS cells after 15 h co-culture may account for the limited effect these bacteria had on iron uptake and distribution. Accordingly, it will be important to understand whether the observed difference in iron homeostasis reflects decreased entry, reduced survival and/or enhanced exit of *H*. *pylori* from their intracellular niche.

In summary, these results provide further evidence that the CagA protein plays a major role in altering host cell iron metabolism in *H*. *pylori*-infected AGS cells, and that this effect is mediated via mechanisms that include increased iron uptake through transferrin endocytosis, and decreased cLIP/increased lysosomal iron through enhanced expression of H-ferritin that are together associated with increased bacterial uptake. We speculate that the increase in lysosomal labile iron observed in *H*. *pylori*-infected AGS cells results in improved growth of *H*. *pylori* and, if proven, this mechanism could help explain *H*. *pylori* persistence in the gastric mucosa.

## Supporting information

S1 Table Primers used for RT-PCR in this study. (DOCX)

S1 Fig Densitometric analysis of sulphide-silver images. Images from sulphide-silver staining were obtained by light microscopy, and treated using the ImageJ software from NIH. Images were first converted into an 8-bit image (A) that was later duplicated and converted to a black and white image (not a grayscale) with cells in black (B). The original and black and white images were compared and mean pixel intensity and standard deviations were obtained for each cell and for the whole cell population present in the image (C).
(TIF)

S2 Fig Total iron measured in AGS cells spiked with *H*. *pylori*. Total intracellular iron was measured by the ferrozine assay in uninfected AGS cells (Ctrl) or the same number of cells spiked with 2 x 106 (+ Hp 2e6), 1 x 107 (+ Hp 1e7), or 2 x 107 (+ Hp 2e7) colony forming units of H. pylori, with protein concentration used to standardize cell numbers. Results are ± SEM of 3 independent experiments. No significant difference was detected between conditions (One-way ANOVA with Tukey’s test for multiple comparisons).
(TIF)

S3 Fig Distribution of transferrin receptor, H-ferritin and lysosomal iron in in AGS cells infected with *H*. *pylori cagA* and *vacA* isogenic mutant strains. AGS cells infected with *H*. *pylori* strain (MOI, 10:1) for 15 h were fixed and stained with Alexa Fluor 488-labelled transferrin receptor (green), Texas Red-conjugated phalloidin-stained actin (red) and Hoechst 33342-stained nuclei (blue) in non-permeabilized cells; Streptavidin-Phycoerythrin-stained H-ferritin (orange) and Hoechst 33342-stained nuclei (blue) in Triton X-100 permeabilized cells, and lysosomal iron using sulphide-silver (left, middle and right columns, respectively). The images, which are representative of three independent experiments, denote cells with *H*. *pylori* 60190 *vacA* (Δ *vacA*) and *cagA* (Δ *cagA*) mutant bacteria. Bar = 50μm.
(TIF)
