# Supplementary material for: Helicobacter pylori infection perturbs iron homeostasis in gastric epithelial cells
Source: PLoS One. 2017 Sep 5;12(9):e0184026. doi: 10.1371/journal.pone.0184026 (PMC5584798; doi:10.1371/journal.pone.0184026)
Supplement: S1 Table — (DOCX) [file pone.0184026.s001.docx]

| **Product** | **Forward primer 5’**→ **3’** | **Reverse primer 5’**→ **3’** | **Ref.** |
| --- | --- | --- | --- |
| TfR | CAGGAACCGAGTCTCCAGTGA | CTTGATGGTGCCGGTGAAGT | (13) |
| H-ferritin | CTCATGAGGAGAGGGAACATGC | CGCTCTCCCAGTCATCACAGT | (14) |
| HPRT | TGACCTTGATTTATTTTGCATACC | CGAGCAAGACGTTCAGTCCT | (20) |

**S1 Table. Primers used for RT-PCR in this study**. TfR, Transferrin receptor; H-ferritin, Ferritin heavy chain; HPRT, Hypoxanthine phosphoribosyltransferase 1.
