# Supplementary figures and images for: Helicobacter pylori infection perturbs iron homeostasis in gastric epithelial cells
Source: PLoS One. 2017 Sep 5;12(9):e0184026. doi: 10.1371/journal.pone.0184026 (PMC5584798; doi:10.1371/journal.pone.0184026)

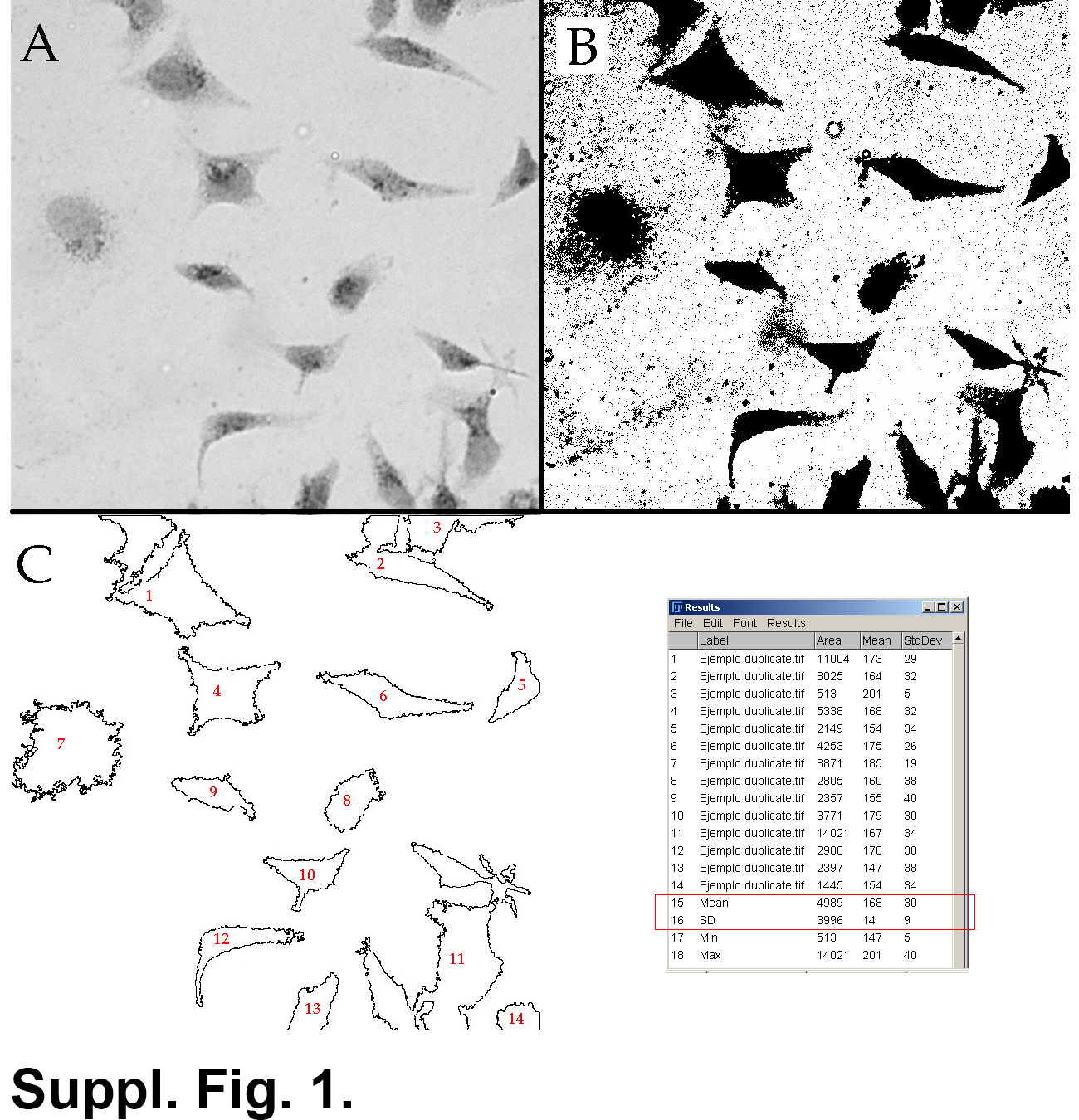

Supplement: S1 Fig — Images from sulphide-silver staining were obtained by light microscopy, and treated using the ImageJ software from NIH. Images were first converted into an 8-bit image (A) that was later duplicated and converted to a black and white image (not a grayscale) with cells in black (B). The original and black and white images were compared and mean pixel intensity and standard deviations were obtained for each cell and for the whole cell population present in the image (C). (TIF) [file pone.0184026.s002.tif]

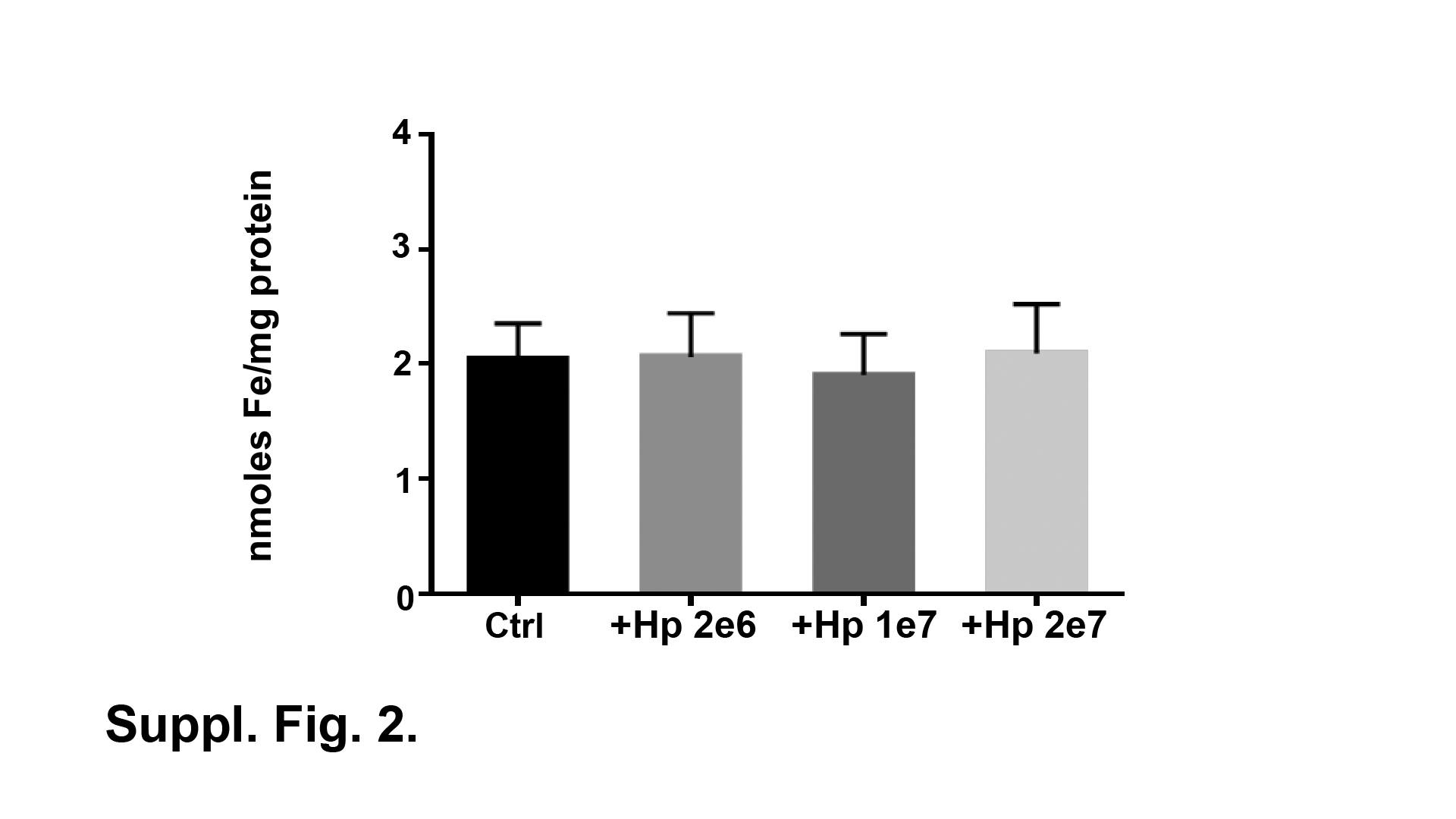

Supplement: S2 Fig — Total intracellular iron was measured by the ferrozine assay in uninfected AGS cells (Ctrl) or the same number of cells spiked with 2 x 10^6^ (+ Hp 2e6), 1 x 10^7^ (+ Hp 1e7), or 2 x 10^7^ (+ Hp 2e7) colony forming units of H. pylori, with protein concentration used to standardize cell numbers. Results are ± SEM of 3 independent experiments. No significant difference was detected between conditions (One-way ANOVA with Tukey’s test for multiple comparisons). (TIF) [file pone.0184026.s003.tif]

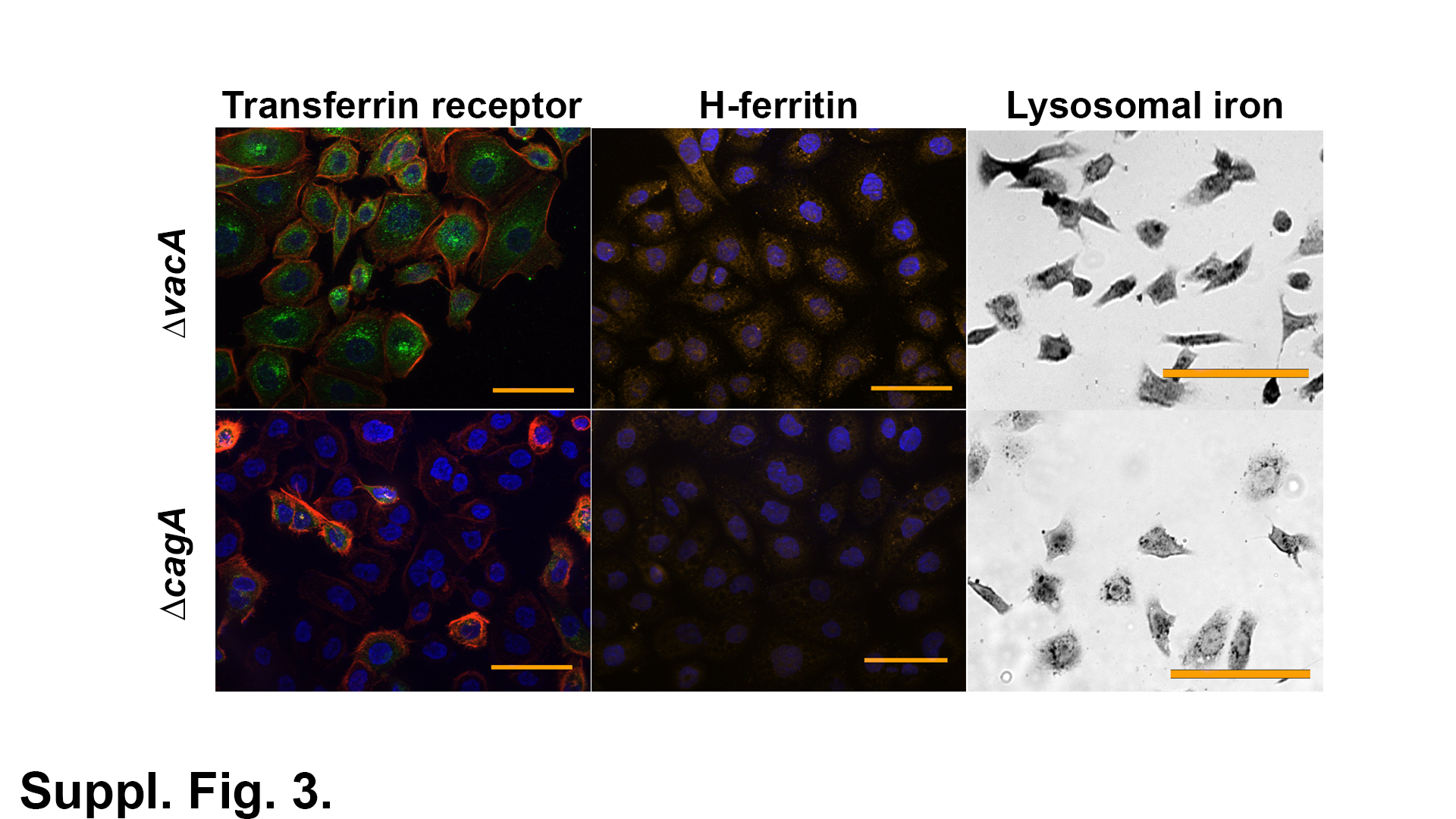

Supplement: S3 Fig — AGS cells infected with H. pylori strain (MOI, 10:1) for 15 h were fixed and stained with Alexa Fluor 488-labelled transferrin receptor (green), Texas Red-conjugated phalloidin-stained actin (red) and Hoechst 33342-stained nuclei (blue) in non-permeabilized cells; Streptavidin-Phycoerythrin-stained H-ferritin (orange) and Hoechst 33342-stained nuclei (blue) in Triton X-100 permeabilized cells, and lysosomal iron using sulphide-silver (left, middle and right columns, respectively). The images, which are representative of three independent experiments, denote cells with H. pylori 60190 vacA (Δ vacA) and cagA (Δ cagA) mutant bacteria. Bar = 50μm. (TIF) [file pone.0184026.s004.tif]
